# Supplementary material for: Deviations in RSV epidemiological patterns and population structures in the United States following the COVID-19 pandemic
Source: Nat Commun. 2024 Apr 20;15:3374. doi: 10.1038/s41467-024-47757-9 (PMC11032338; doi:10.1038/s41467-024-47757-9)
Supplement: Supplementary file 1 — Supplementary Information [file 41467_2024_47757_MOESM1_ESM.pdf]

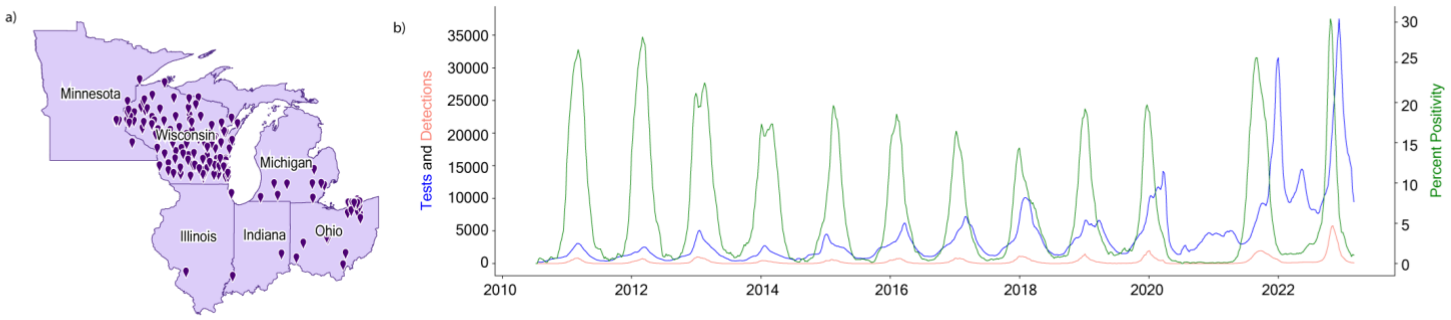

Supplementary Figure 1 | Epidemiology of RSV in the Upper Midwest of the United States. a, Locations of participating laboratories in the Department of Health and Human Services (HHS) Region 5 (Illinois, Indiana, Michigan, Minnesota, Ohio, and Wisconsin) that provide RSV testing data to the National Respiratory and Enteric Virus Surveillance System (NREVSS). **b,** Epidemiology of RSV in HHS Region 5 as represented by a 3-week rolling average of tests (blue), detections (pink), and percent positivity (green) between July 10th, 2010, and June 3rd, 2023 per data provided to the NREVSS.

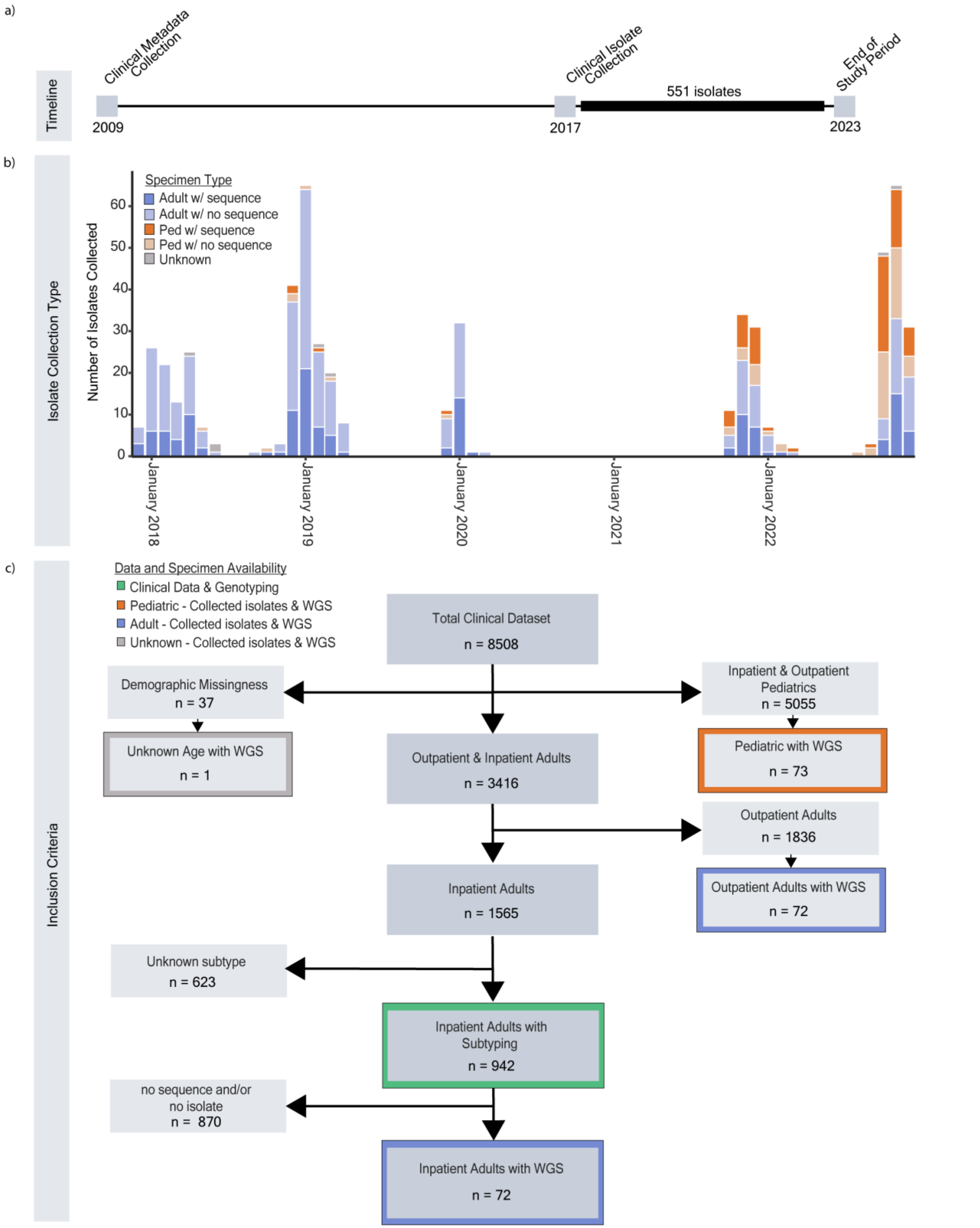

Supplementary Figure 2 | Timeline and Inclusion Criteria of RSV Clinical Metadata and Isolate Collection.

a, Timeline of the study, including clinical metadata and isolate collection. **b,** Absolute counts of isolates collected

over the study period by month. Isolates are categorized by whether they are isolated from adult patients with (dark purple) or without (light purple) sequence information, as well as from pediatric patients with (dark orange) or without (light orange) sequence information. Samples from patients without age information are categorized as unknown (gray). **c,** Inclusion and exclusion criteria of patient metadata and isolates. Patient subsets used for logistic regression modeling (green) or whole genome sequencing (dark purple, orange, and gray) are highlighted.

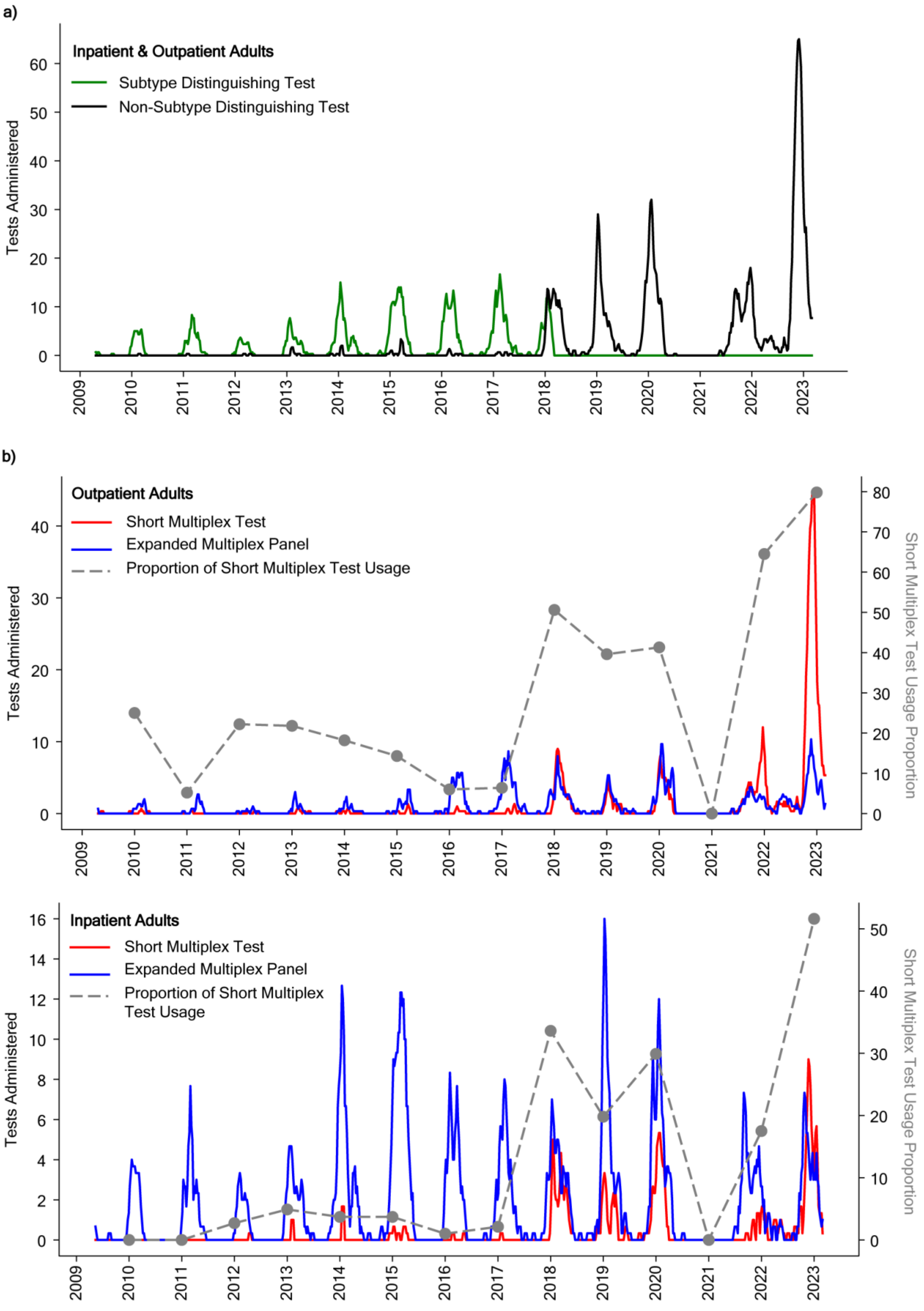
a)
Inpatient & Outpatient Adults
Subtype Distinguishing Test
Non-Subtype Distinguishing Test
Tests Administered
b)
Outpatient Adults
Short Multiplex Test
Expanded Multiplex Panel
Proportion of Short Multiplex Test Usage
Tests Administered
Short Multiplex Test Usage Proportion
Inpatient Adults
Short Multiplex Test
Expanded Multiplex Panel
Proportion of Short Multiplex Test Usage
Tests Administered
Short Multiplex Test Usage Proportion
2009
2010
2011
2012
2013
2014
2015
2016
2017
2018
2019
2020
2021
2022
2023

Supplementary Figure 3 | Shifts in Diagnostic Platform Use among Adult Inpatient and Outpatient Encounters from 2010 to 2023. a, Distribution of RSV subtype-distinguishing diagnostic tests over time as represented by a 3-week rolling average of tests that provide (green, n = 1108) or don't provide (black, n = 2047) subtyping information. **b,** 3-week rolling average of short (red) and expanded, multiplex panel (blue) tests administered among outpatient adults (top, n = 1644) and inpatient adults (bottom, n = 1567). Encounters with unknown diagnostic types and testing categories representative of less than 1% of the dataset (n = 205) were excluded. The proportion of short multiplex diagnostic use (*i.e.*, PCR Test for Influenza and RSV, or Triplex PCR Test for Influenza, RSV, and SARS-CoV-2) for each season is also shown (gray). All reported encounters occurred between April 8^{th,} 2009 to March 1^{st,} 2023.

a)

RSV-A vs. RSV-B Infection<br>full model			
**Characteristic**	**OR[1]**	**95% CI[1]**	**p-value**
Sex			
Female	—	—	
Male	1.23	0.94, 1.61	0.13
Race			
White	—	—	
Asian	0.68	0.30, 1.52	0.3
Black or African American	1.18	0.87, 1.62	0.3
Declined or Unable to Respond	0.88	0.40, 1.96	0.8
Other	0.90	0.56, 1.43	0.6
Ethnicity			
Not Hispanic or Latino	—	—	
Declined or Unable to Respond	0.86	0.39, 1.90	0.7
Hispanic or Latino	0.94	0.58, 1.53	0.8
BMI	0.99	0.98, 1.01	0.3
Age at Admission	1.01	1.00, 1.01	0.11
Comorbidity Sum	0.92	0.85, 0.99	**0.025**
ICU Admission	0.68	0.49, 0.94	**0.018**
RSV associated death	1.33	0.73, 2.45	0.4
Month of Patient Admission	1.00	1.00, 1.00	0.11
Hospitalization Length of Stay	1.01	0.99, 1.02	0.5

[1] OR = Odds Ratio, CI = Confidence Interval

b)

RSV-A vs. RSV-B Infection<br>full model prepandemic			
**Characteristic**	**OR[1]**	**95% CI[1]**	**p-value**
Sex			
Female	—	—	
Male	1.25	0.95, 1.64	0.11
Race			
White	—	—	
Asian	0.68	0.30, 1.53	0.4
Black or African American	1.16	0.85, 1.58	0.4
Declined or Unable to Respond	0.92	0.42, 2.06	0.8
Other	0.91	0.57, 1.46	0.7
Ethnicity			
Not Hispanic or Latino	—	—	
Declined or Unable to Respond	0.89	0.40, 1.98	0.8
Hispanic or Latino	0.97	0.60, 1.58	>0.9
BMI	0.99	0.98, 1.01	0.3
Age at Admission	1.01	1.00, 1.01	0.093
Comorbidity Sum	0.92	0.86, 1.00	**0.040**
ICU Admission	0.68	0.49, 0.95	**0.022**
RSV associated death	1.32	0.72, 2.44	0.4
Month of Patient Admission	1.00	1.00, 1.00	0.2
Hospitalization Length of Stay	1.00	0.99, 1.02	0.6

[1] OR = Odds Ratio, CI = Confidence Interval

c)

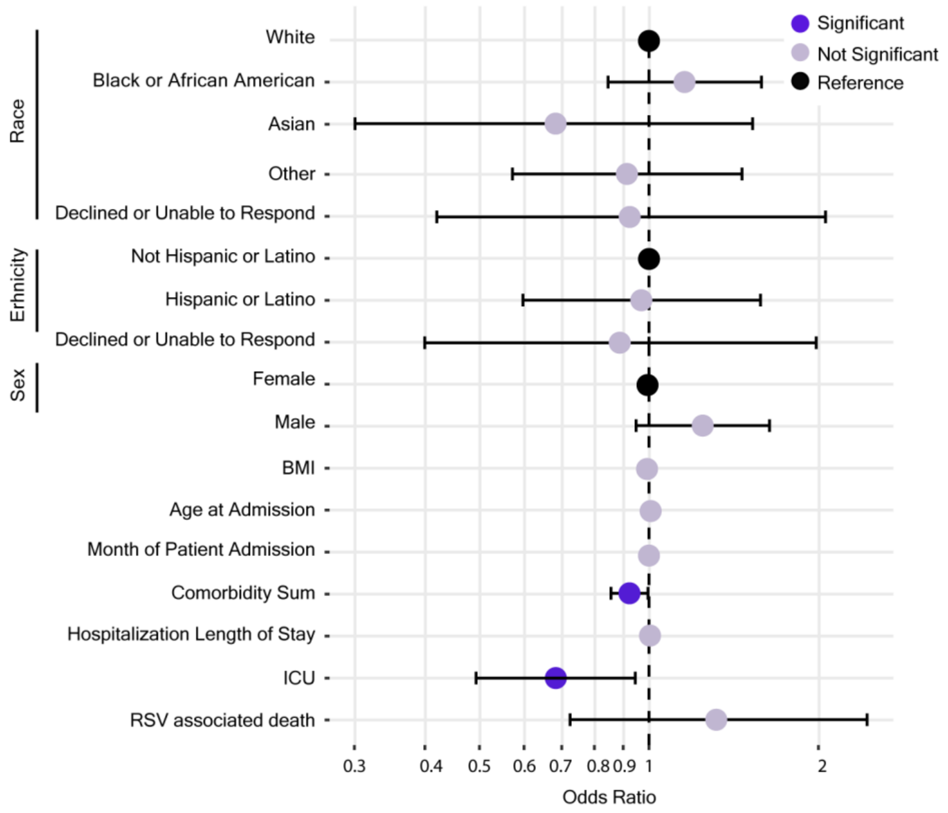

Supplementary Figure 4 | Modeling Inpatient Outcome by RSV Subtype. a, Parameters incorporated in the multivariable logistic regression to model RSV subtype infection among adult inpatients (n = 942). **b,** Parameters incorporated in the multivariable logistic regression to model RSV infection in adult inpatients, excluding the 2020-2021 season and beyond. **c,** Odds ratio plot with 95% confidence intervals (CI) as calculated by a multivariable logistic regression model with RSV-A (reference) or RSV-B infection as the outcome variable in adult inpatients, excluding the 2020-2021 season and beyond. Significant features (p-value < 0.05) are highlighted in dark purple, insignificant features (p-value >= 0.05) are shown in light purple, and reference categories for categorical variables are shown in black.

a)

ICU Admission
full model

Characteristic	OR[1]	95% CI[1]	p-value
Sex			
Female	—	—	
Male	1.38	0.99, 1.93	0.059
Race			
White	—	—	
Asian	1.06	0.37, 2.76	>0.9
Black or African American	1.10	0.74, 1.61	0.6
Declined or Unable to Respond	0.76	0.27, 2.03	0.6
Other	1.17	0.66, 2.05	0.6
Ethnicity			
Not Hispanic or Latino	—	—	
Declined or Unable to Respond	0.71	0.24, 1.93	0.5
Hispanic or Latino	1.04	0.57, 1.87	0.9
RSV Subtype			
RSV-A Infection	—	—	
RSV-B Infection	0.69	0.49, 0.95	**0.025**
BMI	1.01	0.99, 1.03	0.3
Age at Admission	1.01	1.00, 1.02	0.14
Comorbidity Sum	0.96	0.87, 1.05	0.4
RSV associated death	9.98	4.83, 22.1	**<0.001**
Month of Patient Admission	1.00	1.00, 1.00	0.3
Hospitalization Length of Stay	1.13	1.10, 1.16	**<0.001**

[1] OR = Odds Ratio, CI = Confidence Interval

b)

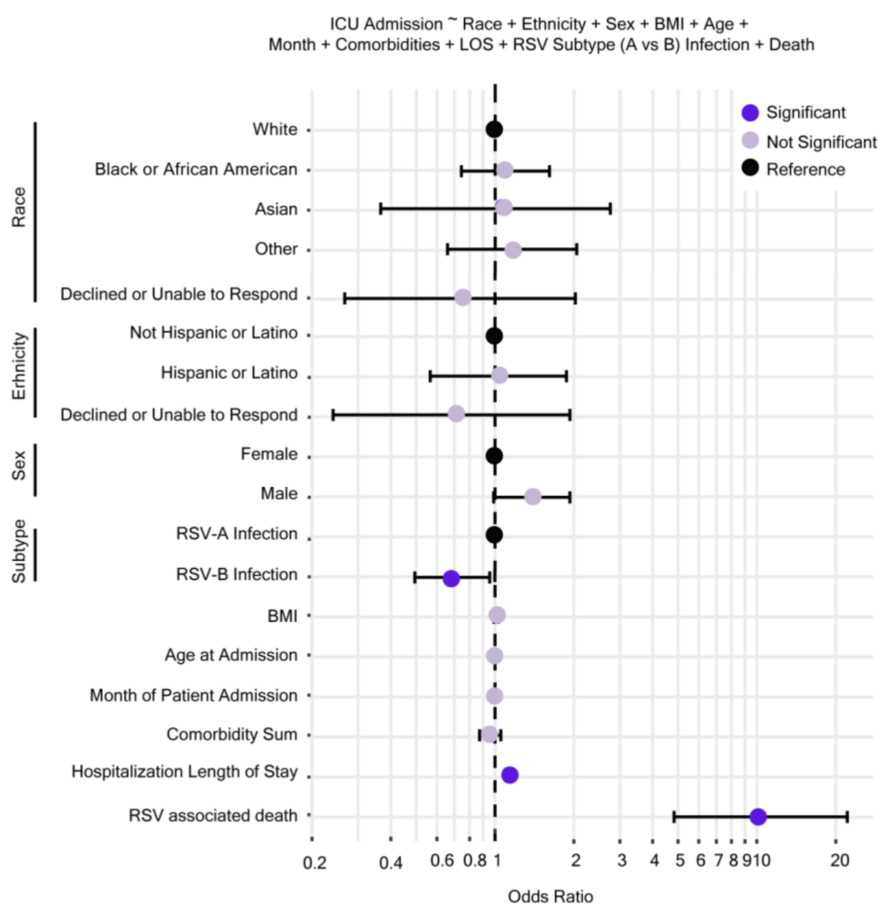

Supplementary Figure 5 | Modeling Inpatient Outcome by ICU Admission. a, Parameters incorporated in the multivariable logistic regression to model ICU admission among adult inpatients (n = 942). **b,** Odds ratio plot with 95% confidence intervals (CI) as calculated by a multivariable logistic regression model with ICU Admission as the outcome variable in adult inpatients. Significant features (p-value < 0.05) are highlighted in dark purple, insignificant features (p-value >= 0.05) are shown in light purple, and reference categories for categorical variables are shown in black.

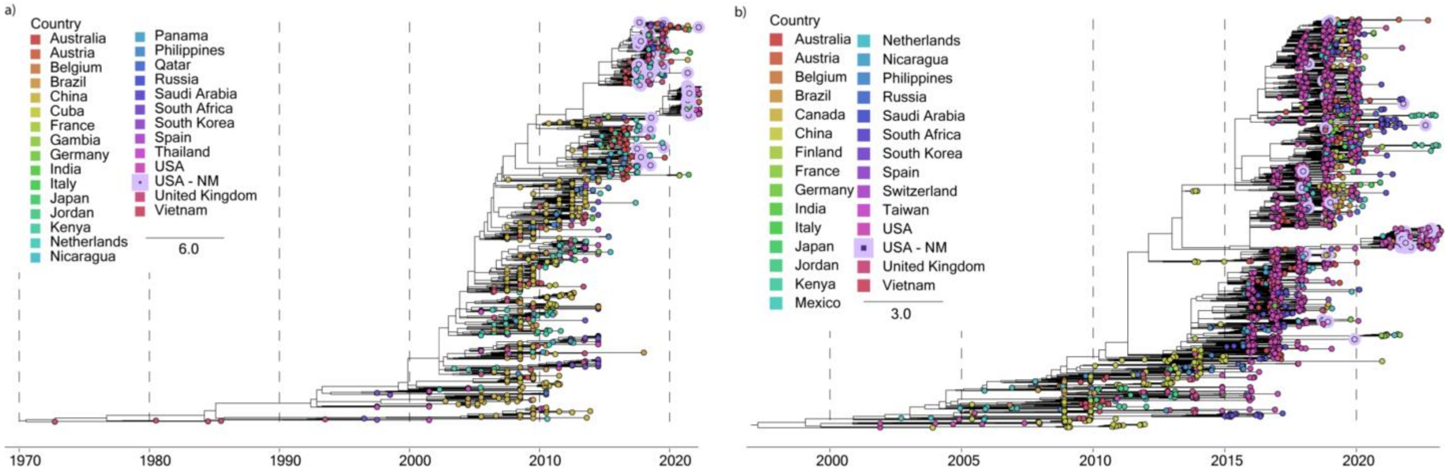

Supplementary Figure 6 | Temporal Analysis of RSV-B F and G. a, Maximum likelihood (ML) phylogenetic temporal tree of unique circulating RSV-B complete G sequence collected from 1976 to 2023 as of October 2023 (n = 983). **b,** ML phylogenetic temporal tree of unique circulating RSV-B complete F sequences collected from 1997 to 2023 as of October 2023 (n= 2067). Branch tips are colored by country of origin, and scale units are substitutions/site/year.

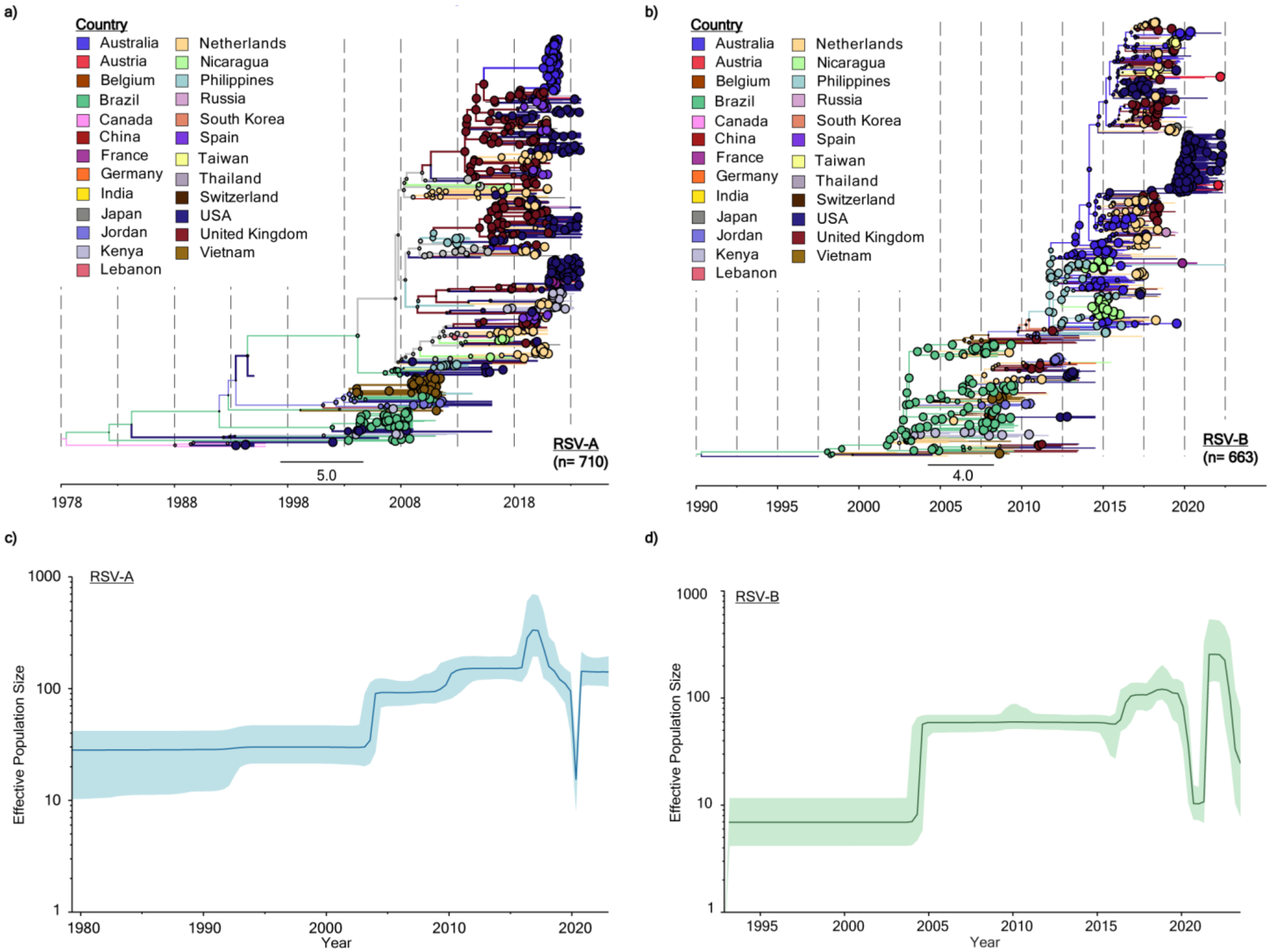

Supplementary Figure 7 | Global Phylogenetic Analysis of RSV-A and RSV-B. Bayesian phylogenetic temporal tree of circulating **a,** RSV-A (excluding clade's A.2, A.1.1, & A.2.1.1) (n = 710) and **b,** all RSV-B (n = 663) complete genome sequences as of May 2023. Each phylogenetic tree corresponds to the maximum clade credibility tree by depicting branch color by the most probable geographical location (*i.e.*, country) of their descendent node. Node circle size corresponds to the probability of probable geographical location defining the nod. **c,** RSV-A and **d,** RSV-B population dynamics using Bayesian skyline analyses to show effective population size with respect to time. Shadings in each plot represent the 95% highest posterior density (HPD).

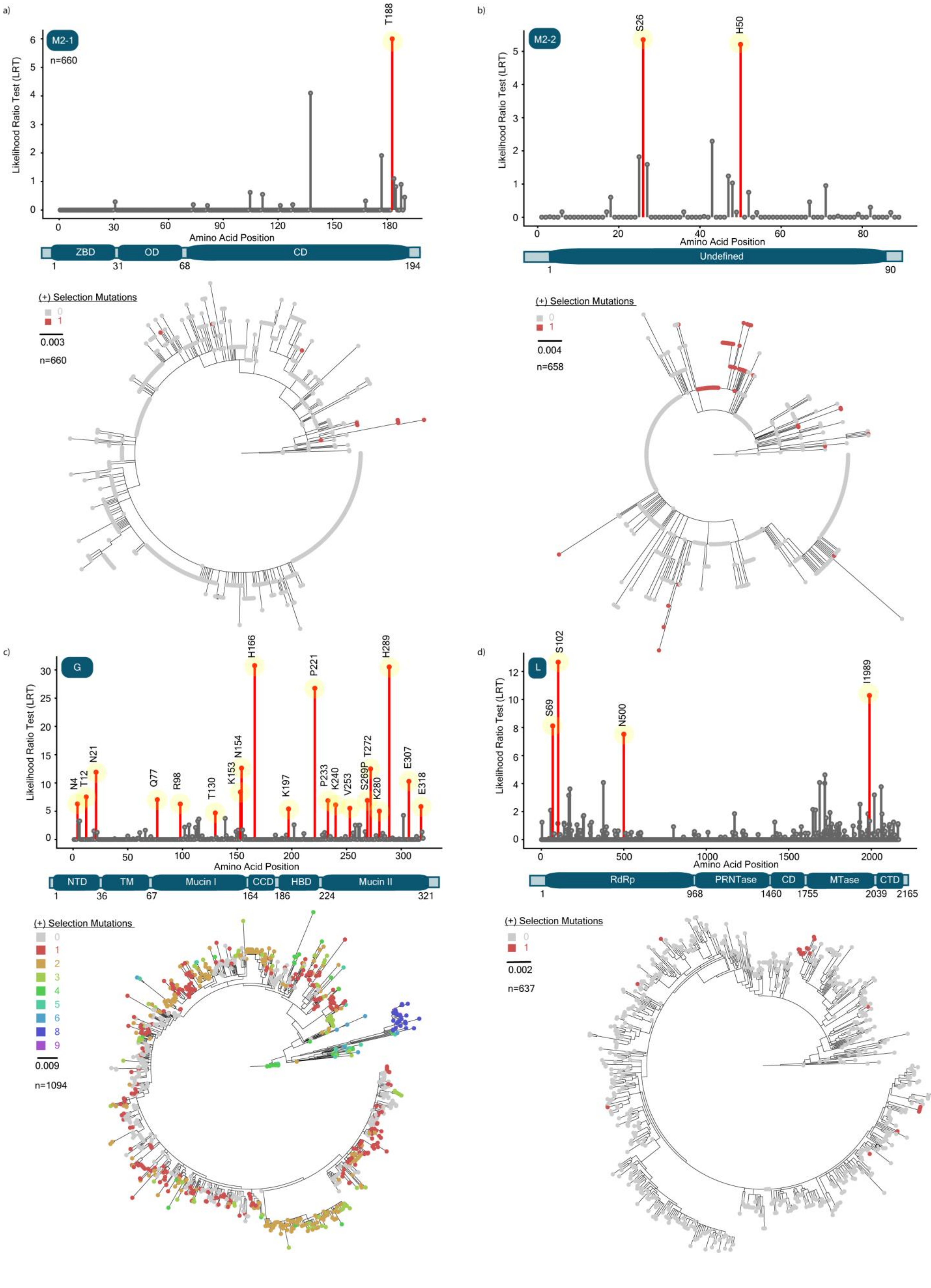

a)
M2-1
n=660
T188
Likelihood Ratio Test (LRT)
Amino Acid Position
ZBD
OD
CD
1
31
68
194
(+) Selection Mutations
0
1
0.003
n=660
b)
M2-2
S26
H50
Likelihood Ratio Test (LRT)
Amino Acid Position
Undefined
1
90
(+) Selection Mutations
0
1
0.004
n=658
c)
G
N4
T12
N21
Q77
R98
T130
K153
N154
H166
K197
P221
P233
K240
V253
S269P
T272
K280
H289
E307
E318
Likelihood Ratio Test (LRT)
Amino Acid Position
NTD
TM
Mucin I
CCD
HBD
Mucin II
1
36
67
164
186
224
321
(+) Selection Mutations
0
1
2
3
4
5
6
8
9
0.009
n=1094
d)
L
S69
S102
N500
I1989
Likelihood Ratio Test (LRT)
Amino Acid Position
RdRp
PRNTase
CD
MTase
CTD
1
968
1460
1755
2039
2165
(+) Selection Mutations
0
1
0.002
n=637

Supplementary Figure 8 | Positive Selection of RSV-B ORFs. Lollipop plots (top) for unique RSV-B open reading frames (ORFs) sequences with statistically significant sites of positive selection. Amino acid positions with statistically significant likelihood ratio test (LRT) (p-value < 0.05) indicating episodic selection by a mixed effects model of evolution (MEME) are colored in red and annotated using a generated most recent common ancestor (MRCA). Nonsignificant LRT and posterior probability are in gray. Maximum likelihood (ML) genetic diversity trees (bottom) for ORFs depicted in lollipop plots to display the isolates that contain mutations at positive selection sites. Branch tips are colored by the number of mutations present at positively selected sites. The ORF sequences used are globally sampled from 1957 to 2023 for a, M2-1 (n = 660), b, M2-2 (n. = 658), c, G (n = 1094), and d, L (n = 637).

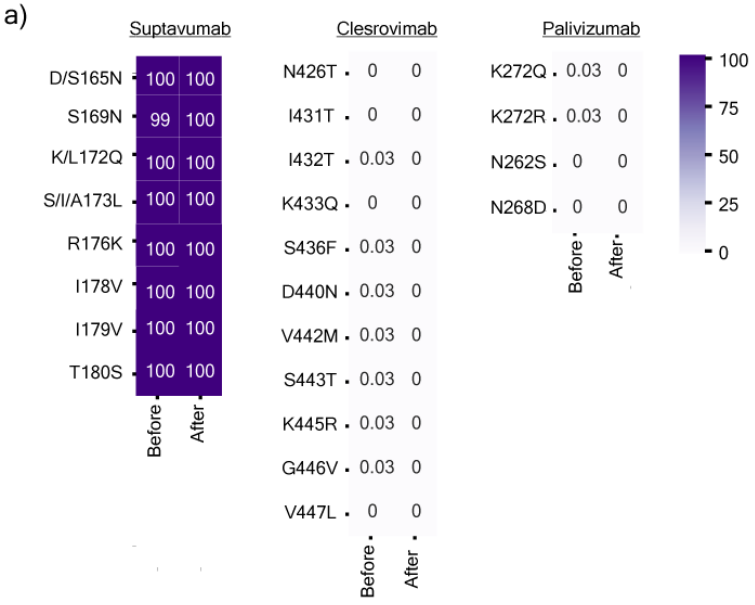

Supplementary Figure 9 | Globally Circulating RSV-B Mutations in Fusion Protein on Mab Binding Site

a**,** Heatmap displaying mutational frequencies at positions within the Suptavumab, Clesrovimab, and Palivizumab binding site before and after May 1, 2020, as compared to generated most-recent-common ancestor (MRCA).

NCBI ID	Nextstrain clade	G clade	Accession Number
RSV-A/human/USA/IL-NM-RSV012/2019	A.D.1	GA2.3.5	PP352278
RSV-A/human/USA/IL-NM-RSV013/2019	A.D.1	GA2.3.5	PP352279
RSV-A/human/USA/IL-NM-RSV016/2019	A.D.1	GA2.3.5	PP352280
RSV-A/human/USA/IL-NM-RSV026/2018	A.D.1	GA2.3.5	PP352281
RSV-A/human/USA/IL-NM-RSV044/2019	A.D.2.3	GA2.3.5	PP352282
RSV-A/human/USA/IL-NM-RSV045/2019	A.D.1	GA2.3.5	PP352283
RSV-A/human/USA/IL-NM-RSV046/2019	A.D.2.2	GA2.3.5	PP352284
RSV-A/human/USA/IL-NM-RSV066/2019	A.D.1	GA2.3.5	PP352285
RSV-A/human/USA/IL-NM-RSV069/2019	A.D.3	GA2.3.5	PP352286
RSV-A/human/USA/IL-NM-RSV078/2018	A.D.4	GA2.3.5	PP352287
RSV-A/human/USA/IL-NM-RSV088/2018	A.D.3	GA2.3.5	PP352288
RSV-A/human/USA/IL-NM-RSV091/2018	A.D.1	GA2.3.5	PP352289
RSV-A/human/USA/IL-NM-RSV094/2019	A.D.1	GA2.3.5	PP352290
RSV-A/human/USA/IL-NM-RSV095/2019	A.D.2.2	GA2.3.5	PP352291
RSV-A/human/USA/IL-NM-RSV103/2019	A.D.3	GA2.3.5	PP352292
RSV-A/human/USA/IL-NM-RSV108/2018	A.D	GA2.3.5	PP352293
RSV-A/human/USA/IL-NM-RSV110/2019	A.D.1	GA2.3.5	PP352294
RSV-A/human/USA/IL-NM-RSV124/2019	A.D.1	GA2.3.5	PP352295
RSV-A/human/USA/IL-NM-RSV128/2019	A.D.4	GA2.3.5	PP352296
RSV-A/human/USA/IL-NM-RSV130/2019	A.D.1	GA2.3.5	PP352297
RSV-A/human/USA/IL-NM-RSV148/2019	A.D.2.3	GA2.3.5	PP352298
RSV-A/human/USA/IL-NM-RSV159/2019	A.D.2.3	GA2.3.5	PP352299
RSV-A/human/USA/IL-NM-RSV162/2019	A.D.4	GA2.3.5	PP352300
RSV-A/human/USA/IL-NM-RSV175/2018	A.D.1	GA2.3.5	PP352301
RSV-A/human/USA/IL-NM-RSV180/2018	A.D.4	GA2.3.5	PP352302
RSV-A/human/USA/IL-NM-RSV181/2018	A.D.1	GA2.3.5	PP352303
RSV-A/human/USA/IL-NM-RSV182/2018	A.D.2.2	GA2.3.5	PP352304
RSV-A/human/USA/IL-NM-RSV187/2018	A.D.1	GA2.3.5	PP352305
RSV-A/human/USA/IL-NM-RSV188/2018	A.D.1	GA2.3.5	PP352306
RSV-A/human/USA/IL-NM-RSV194/2019	A.D.3	GA2.3.5	PP352307
RSV-A/human/USA/IL-NM-RSV199/2019	A.D.1	GA2.3.5	PP352308
RSV-A/human/USA/IL-NM-RSV200/2019	A.D	GA2.3.5	PP352309
RSV-A/human/USA/IL-NM-RSV207/2020	A.D.1	GA2.3.5	PP352310
RSV-A/human/USA/IL-NM-RSV209/2020	A.D.1	GA2.3.5	PP352311
RSV-A/human/USA/IL-NM-RSV210/2020	A.D.1	GA2.3.5	PP352312
RSV-A/human/USA/IL-NM-RSV215/2020	A.D.3	GA2.3.5	PP352313
RSV-A/human/USA/IL-NM-RSV216/2020	A.D.3	GA2.3.5	PP352314
RSV-A/human/USA/IL-NM-RSV218/2019	A.D.1	GA2.3.5	PP352315
RSV-A/human/USA/IL-NM-RSV224/2020	A.D.1	GA2.3.5	PP352316
RSV-A/human/USA/IL-NM-RSV228/2020	A.D.2	GA2.3.5	PP352317
RSV-A/human/USA/IL-NM-RSV231/2020	A.D.5	GA2.3.5	PP352318
RSV-A/human/USA/IL-NM-RSV241/2019	A.D.1	GA2.3.5	PP352319
RSV-A/human/USA/IL-NM-RSV294/2018	A.D.5	GA2.3.5	PP352320
RSV-A/human/USA/IL-NM-RSV300/2019	A.D.2.2	GA2.3.5	PP352321
RSV-A/human/USA/IL-NM-RSV302/2019	A.D.1	GA2.3.5	PP352322
RSV-A/human/USA/IL-NM-RSV336/2021	A.D.3	GA2.3.5	PP352323
RSV-A/human/USA/IL-NM-RSV378/2021	A.D.1	GA2.3.5	PP352324
RSV-A/human/USA/IL-NM-RSV403/2022	A.D.1	GA2.3.5	PP352325
RSV-A/human/USA/IL-NM-RSV404/2022	A.D.1	GA2.3.5	PP352326
RSV-A/human/USA/IL-NM-RSV408/2022	A.D.1	GA2.3.5	PP352327

RSV-A/human/USA/IL-NM-RSV409/2022	A.D.5.2	GA2.3.5	PP352328
RSV-A/human/USA/IL-NM-RSV410/2022	A.D.5.1	GA2.3.5	PP352329
RSV-A/human/USA/IL-NM-RSV411/2022	A.D.1	GA2.3.5	PP352330
RSV-A/human/USA/IL-NM-RSV414/2022	A.D.5.2	GA2.3.5	PP352331
RSV-A/human/USA/IL-NM-RSV415/2022	A.D.5.2	GA2.3.5	PP352332
RSV-A/human/USA/IL-NM-RSV416/2022	A.D.1	GA2.3.5	PP352333
RSV-A/human/USA/IL-NM-RSV417/2022	A.D.3	GA2.3.5	PP352334
RSV-A/human/USA/IL-NM-RSV418/2022	A.D.5.2	GA2.3.5	PP352335
RSV-A/human/USA/IL-NM-RSV421/2022	A.D.5.2	GA2.3.5	PP352336
RSV-A/human/USA/IL-NM-RSV422/2022	A.D.3	GA2.3.5	PP352337
RSV-A/human/USA/IL-NM-RSV424/2022	A.D.1	GA2.3.5	PP352338
RSV-A/human/USA/IL-NM-RSV427/2022	A.D.5.2	GA2.3.5	PP352339
RSV-A/human/USA/IL-NM-RSV428/2022	A.D.1	GA2.3.5	PP352340
RSV-A/human/USA/IL-NM-RSV429/2022	A.D.1	GA2.3.5	PP352341
RSV-A/human/USA/IL-NM-RSV430/2022	A.D.1	GA2.3.5	PP352342
RSV-A/human/USA/IL-NM-RSV431/2022	A.D.5.2	GA2.3.5	PP352343
RSV-A/human/USA/IL-NM-RSV434/2022	A.D.1	GA2.3.5	PP352344
RSV-A/human/USA/IL-NM-RSV435/2022	A.D.1	GA2.3.5	PP352345
RSV-A/human/USA/IL-NM-RSV441/2022	A.D.3	GA2.3.5	PP352346
RSV-A/human/USA/IL-NM-RSV443/2022	A.D.1	GA2.3.5	PP352347
RSV-A/human/USA/IL-NM-RSV447/2022	A.D.5.2	GA2.3.5	PP352348
RSV-A/human/USA/IL-NM-RSV448/2022	A.D.1	GA2.3.5	PP352349
RSV-A/human/USA/IL-NM-RSV451/2022	A.D.1	GA2.3.5	PP352350
RSV-A/human/USA/IL-NM-RSV452/2022	A.D.1	GA2.3.5	PP352351
RSV-A/human/USA/IL-NM-RSV454/2022	A.D.1	GA2.3.5	PP352352
RSV-A/human/USA/IL-NM-RSV456/2022	A.D.3	GA2.3.5	PP352353
RSV-A/human/USA/IL-NM-RSV457/2022	A.D.1	GA2.3.5	PP352354
RSV-A/human/USA/IL-NM-RSV464/2022	A.D.3	GA2.3.5	PP352355
RSV-A/human/USA/IL-NM-RSV465/2022	A.D.1	GA2.3.5	PP352356
RSV-A/human/USA/IL-NM-RSV466/2022	A.D.1	GA2.3.5	PP352357
RSV-A/human/USA/IL-NM-RSV468/2022	A.D.5.1	GA2.3.5	PP352358
RSV-A/human/USA/IL-NM-RSV471/2022	A.D.5.2	GA2.3.5	PP352359
RSV-A/human/USA/IL-NM-RSV475/2022	A.D.5.2	GA2.3.5	PP352360
RSV-A/human/USA/IL-NM-RSV480/2022	A.D.1	GA2.3.5	PP352361
RSV-A/human/USA/IL-NM-RSV483/2022	A.D.1	GA2.3.5	PP352362
RSV-A/human/USA/IL-NM-RSV484/2022	A.D.3	GA2.3.5	PP352363
RSV-A/human/USA/IL-NM-RSV485/2022	A.D.3	GA2.3.5	PP352364
RSV-A/human/USA/IL-NM-RSV486/2022	A.D.5.2	GA2.3.5	PP352365
RSV-A/human/USA/IL-NM-RSV489/2022	A.D.5.2	GA2.3.5	PP352366
RSV-A/human/USA/IL-NM-RSV490/2022	A.D.3.1	GA2.3.5	PP352367
RSV-A/human/USA/IL-NM-RSV494/2022	A.D.1	GA2.3.5	PP352368
RSV-A/human/USA/IL-NM-RSV495/2022	A.D.1	GA2.3.5	PP352369
RSV-A/human/USA/IL-NM-RSV501/2022	A.D.5.2	GA2.3.5	PP352370
RSV-A/human/USA/IL-NM-RSV504/2022	A.D.3	GA2.3.5	PP352371
RSV-A/human/USA/IL-NM-RSV505/2022	A.D.5.2	GA2.3.5	PP352372
RSV-A/human/USA/IL-NM-RSV508/2022	A.D.5.2	GA2.3.5	PP352373
RSV-A/human/USA/IL-NM-RSV511/2022	A.D.5.2	GA2.3.5	PP352374
RSV-A/human/USA/IL-NM-RSV513/2022	A.D.1	GA2.3.5	PP352375
RSV-A/human/USA/IL-NM-RSV515/2022	A.D.1	GA2.3.5	PP352376
RSV-A/human/USA/IL-NM-RSV518/2022	A.D.1	GA2.3.5	PP352377
RSV-A/human/USA/IL-NM-RSV524/2022	A.D.3	GA2.3.5	PP352378

RSV-A/human/USA/IL-NM-RSV527/2022	A.D.3	GA2.3.5	PP352379
RSV-A/human/USA/IL-NM-RSV528/2022	A.D.5.2	GA2.3.5	PP352380
RSV-A/human/USA/IL-NM-RSV529/2022	A.D.3	GA2.3.5	PP352381
RSV-A/human/USA/IL-NM-RSV538/2022	A.D.3	GA2.3.5	PP352382
RSV-A/human/USA/IL-NM-RSV541/2022	A.D.5.2	GA2.3.5	PP352383
RSV-A/human/USA/IL-NM-RSV542/2022	A.D.1	GA2.3.5	PP352384
RSV-A/human/USA/IL-NM-RSV545/2022	A.D.3	GA2.3.5	PP352385
RSV-A/human/USA/IL-NM-RSV547/2022	A.D.3	GA2.3.5	PP352386
RSV-A/human/USA/IL-NM-RSV548/2022	A.D.5.2	GA2.3.5	PP352387
RSV-B/human/USA/IL-NM-RSV015/2018	B.D.5.2.1	GB5.0.5a	PP352388
RSV-B/human/USA/IL-NM-RSV021/2019	B.D.5.2.1	GB5.0.5a	PP352389
RSV-B/human/USA/IL-NM-RSV024/2018	B.D.5.2.1	GB5.0.5a	PP352390
RSV-B/human/USA/IL-NM-RSV039/2018	B.D.5.2.1	GB5.0.5a	PP352391
RSV-B/human/USA/IL-NM-RSV053/2018	B.D.5.2	GB5.0.5a	PP352392
RSV-B/human/USA/IL-NM-RSV055/2019	B.D.5.2.1	GB5.0.5a	PP352393
RSV-B/human/USA/IL-NM-RSV067/2019	B.D.5.2	GB5.0.5a	PP352394
RSV-B/human/USA/IL-NM-RSV081/2019	B.D.5.2	GB5.0.5a	PP352395
RSV-B/human/USA/IL-NM-RSV085/2018	B.D.5.2.1	GB5.0.5a	PP352396
RSV-B/human/USA/IL-NM-RSV106/2017	B.D.5.2	GB5.0.5a	PP352397
RSV-B/human/USA/IL-NM-RSV109/2018	B.D.5.2.1	GB5.0.5a	PP352398
RSV-B/human/USA/IL-NM-RSV111/2017	B.D.5.2.1	GB5.0.5a	PP352399
RSV-B/human/USA/IL-NM-RSV112/2018	B.D.5.2.1	GB5.0.5a	PP352400
RSV-B/human/USA/IL-NM-RSV113/2018	B.D.5.2.1	GB5.0.5a	PP352401
RSV-B/human/USA/IL-NM-RSV115/2017	B.D.5.2.1	GB5.0.5a	PP352402
RSV-B/human/USA/IL-NM-RSV127/2018	B.D.5.2.1	GB5.0.5a	PP352403
RSV-B/human/USA/IL-NM-RSV137/2018	B.D.5.2.1	GB5.0.5a	PP352404
RSV-B/human/USA/IL-NM-RSV140/2018	B.D.5.2.1	GB5.0.5a	PP352405
RSV-B/human/USA/IL-NM-RSV141/2018	B.D.5.2	GB5.0.5a	PP352406
RSV-B/human/USA/IL-NM-RSV142/2019	B.D.5.2.1	GB5.0.5a	PP352407
RSV-B/human/USA/IL-NM-RSV143/2018	B.D.5.2.1	GB5.0.5a	PP352408
RSV-B/human/USA/IL-NM-RSV145/2018	B.D.5.2.1	GB5.0.5a	PP352409
RSV-B/human/USA/IL-NM-RSV149/2019	B.D.5.2.1	GB5.0.5a	PP352410
RSV-B/human/USA/IL-NM-RSV152/2019	B.D.5.2.1	GB5.0.5a	PP352411
RSV-B/human/USA/IL-NM-RSV163/2018	B.D.5.2	GB5.0.5a	PP352412
RSV-B/human/USA/IL-NM-RSV164/2019	B.D.5.2.1	GB5.0.5a	PP352413
RSV-B/human/USA/IL-NM-RSV168/2018	B.D.5.2.1	GB5.0.5a	PP352414
RSV-B/human/USA/IL-NM-RSV171/2018	B.D.5.2.1	GB5.0.5a	PP352415
RSV-B/human/USA/IL-NM-RSV177/2018	B.D.5.2.1	GB5.0.5a	PP352416
RSV-B/human/USA/IL-NM-RSV185/2018	B.D.5.2	GB5.0.5a	PP352417
RSV-B/human/USA/IL-NM-RSV190/2018	B.D.5.2.1	GB5.0.5a	PP352418
RSV-B/human/USA/IL-NM-RSV203/2020	B.D.5.2.1	GB5.0.5a	PP352419
RSV-B/human/USA/IL-NM-RSV208/2020	B.D.5.2.1	GB5.0.5a	PP352420
RSV-B/human/USA/IL-NM-RSV214/2019	B.D.5.2.1	GB5.0.5a	PP352421
RSV-B/human/USA/IL-NM-RSV222/2020	B.D.5.2	GB5.0.5a	PP352422
RSV-B/human/USA/IL-NM-RSV235/2020	B.D.5.2.1	GB5.0.5a	PP352423
RSV-B/human/USA/IL-NM-RSV246/2019	B.D.5.2.1	GB5.0.5a	PP352424
RSV-B/human/USA/IL-NM-RSV247/2018	B.D.5.2.1	GB5.0.5a	PP352425
RSV-B/human/USA/IL-NM-RSV250/2020	B.D.5.2.1	GB5.0.5a	PP352426
RSV-B/human/USA/IL-NM-RSV252/2020	B.D.5.2.1	GB5.0.5a	PP352427

RSV-B/human/USA/IL-NM-RSV253/2019	B.D.5.2.1	GB5.0.5a	PP352428
RSV-B/human/USA/IL-NM-RSV255/2019	B.D.5.2.1	GB5.0.5a	PP352429
RSV-B/human/USA/IL-NM-RSV256/2020	B.D.5.2.1	GB5.0.5a	PP352430
RSV-B/human/USA/IL-NM-RSV260/2018	B.D.5.2.1	GB5.0.5a	PP352431
RSV-B/human/USA/IL-NM-RSV267/2019	B.D.5.2.1	GB5.0.5a	PP352432
RSV-B/human/USA/IL-NM-RSV272/2019	B.D.5.2.1	GB5.0.5a	PP352433
RSV-B/human/USA/IL-NM-RSV280/2018	B.D.5.2.1	GB5.0.5a	PP352434
RSV-B/human/USA/IL-NM-RSV292/2018	B.D.5.2.1	GB5.0.5a	PP352435
RSV-B/human/USA/IL-NM-RSV296/2018	B.D.5.2.1	GB5.0.5a	PP352436
RSV-B/human/USA/IL-NM-RSV298/2019	B.D.5.2.1	GB5.0.5a	PP352437
RSV-B/human/USA/IL-NM-RSV299/2018	B.D.5.2	GB5.0.5a	PP352438
RSV-B/human/USA/IL-NM-RSV303/2018	B.D.5.2.1	GB5.0.5a	PP352439
RSV-B/human/USA/IL-NM-RSV310/2018	B.D.5.2.1	GB5.0.5a	PP352440
RSV-B/human/USA/IL-NM-RSV311/2019	B.D.5.2.1	GB5.0.5a	PP352441
RSV-B/human/USA/IL-NM-RSV313/2021	B.D.5.2.1.1	GB5.0.5a	PP352442
RSV-B/human/USA/IL-NM-RSV314/2021	B.D.5.2.1.1	GB5.0.5a	PP352443
RSV-B/human/USA/IL-NM-RSV316/2021	B.D.5.2.1.1	GB5.0.5a	PP352444
RSV-B/human/USA/IL-NM-RSV317/2021	B.D.5.2.1.1	GB5.0.5a	PP352445
RSV-B/human/USA/IL-NM-RSV320/2021	B.D.5.2.1.1	GB5.0.5a	PP352446
RSV-B/human/USA/IL-NM-RSV321/2021	B.D.5.2.1.1	GB5.0.5a	PP352447
RSV-B/human/USA/IL-NM-RSV323/2021	B.D.5.2.1.1	GB5.0.5a	PP352448
RSV-B/human/USA/IL-NM-RSV324/2021	B.D.5.2.1.1	GB5.0.5a	PP352449
RSV-B/human/USA/IL-NM-RSV326/2021	B.D.5.2.1.1	GB5.0.5a	PP352450
RSV-B/human/USA/IL-NM-RSV327/2021	B.D.5.2.1.1	GB5.0.5a	PP352451
RSV-B/human/USA/IL-NM-RSV329/2021	B.D.5.2.1.1	GB5.0.5a	PP352452
RSV-B/human/USA/IL-NM-RSV333/2021	B.D.5.2.1.1	GB5.0.5a	PP352453
RSV-B/human/USA/IL-NM-RSV335/2021	B.D.5.2.1.1	GB5.0.5a	PP352454
RSV-B/human/USA/IL-NM-RSV337/2021	B.D.5.2.1.1	GB5.0.5a	PP352455
RSV-B/human/USA/IL-NM-RSV340/2021	B.D.5.2.1	GB5.0.5a	PP352456
RSV-B/human/USA/IL-NM-RSV341/2021	B.D.5.2.1.1	GB5.0.5a	PP352457
RSV-B/human/USA/IL-NM-RSV342/2021	B.D.5.2.1.1	GB5.0.5a	PP352458
RSV-B/human/USA/IL-NM-RSV345/2021	B.D.5.2.1.1	GB5.0.5a	PP352459
RSV-B/human/USA/IL-NM-RSV347/2021	B.D.5.2.1.1	GB5.0.5a	PP352460
RSV-B/human/USA/IL-NM-RSV348/2022	B.D.5.2.1.1	GB5.0.5a	PP352461
RSV-B/human/USA/IL-NM-RSV351/2021	B.D.5.2.1.1	GB5.0.5a	PP352462
RSV-B/human/USA/IL-NM-RSV353/2021	B.D.5.2.1.1	GB5.0.5a	PP352463
RSV-B/human/USA/IL-NM-RSV355/2021	B.D.5.2.1.1	GB5.0.5a	PP352464
RSV-B/human/USA/IL-NM-RSV356/2021	B.D.5.2.1.1	GB5.0.5a	PP352465
RSV-B/human/USA/IL-NM-RSV357/2021	B.D.5.2.1.1	GB5.0.5a	PP352466
RSV-B/human/USA/IL-NM-RSV358/2021	B.D.5.2.1.1	GB5.0.5a	PP352467
RSV-B/human/USA/IL-NM-RSV360/2021	B.D.E.1	GB5.0.5a	PP352468
RSV-B/human/USA/IL-NM-RSV361/2021	B.D.5.2.1.1	GB5.0.5a	PP352469
RSV-B/human/USA/IL-NM-RSV366/2021	B.D.5.2.1.1	GB5.0.5a	PP352470
RSV-B/human/USA/IL-NM-RSV368/2021	B.D.5.2.1.1	GB5.0.5a	PP352471
RSV-B/human/USA/IL-NM-RSV370/2021	B.D.5.2.1.1	GB5.0.5a	PP352472
RSV-B/human/USA/IL-NM-RSV373/2021	B.D.5.2.1.1	GB5.0.5a	PP352473
RSV-B/human/USA/IL-NM-RSV374/2021	B.D.5.2.1.1	GB5.0.5a	PP352474
RSV-B/human/USA/IL-NM-RSV375/2021	B.D.5.2.1.1	GB5.0.5a	PP352475
RSV-B/human/USA/IL-NM-RSV379/2021	B.D.5.2.1.1	GB5.0.5a	PP352476

RSV-B/human/USA/IL-NM-RSV381/2021	B.D.5.2.1.1	GB5.0.5a	PP352477
RSV-B/human/USA/IL-NM-RSV382/2022	B.D.5.2.1.1	GB5.0.5a	PP352478
RSV-B/human/USA/IL-NM-RSV383/2021	B.D.5.2.1.1	GB5.0.5a	PP352479
RSV-B/human/USA/IL-NM-RSV385/2021	B.D.5.2.1.1	GB5.0.5a	PP352480
RSV-B/human/USA/IL-NM-RSV387/2021	B.D.5.2.1.1	GB5.0.5a	PP352481
RSV-B/human/USA/IL-NM-RSV389/2021	B.D.5.2.1.1	GB5.0.5a	PP352482
RSV-B/human/USA/IL-NM-RSV394/2021	B.D.5.2.1.1	GB5.0.5a	PP352483
RSV-B/human/USA/IL-NM-RSV400/2022	B.D.5.2.1.1	GB5.0.5a	PP352484
RSV-B/human/USA/IL-NM-RSV401/2022	B.D.5.2.1.1	GB5.0.5a	PP352485
RSV-B/human/USA/IL-NM-RSV446/2022	B.D.5.2.1.4	GB5.0.5a	PP352488
RSV-B/human/USA/IL-NM-RSV458/2022	B.D.5.2.1.1	GB5.0.5a	PP352494
RSV-B/human/USA/IL-NM-RSV463/2022	B.D.5.2.1.1	GB5.0.5a	PP352489
RSV-B/human/USA/IL-NM-RSV476/2022	B.D.5.2.1.1	GB5.0.5a	PP352495
RSV-B/human/USA/IL-NM-RSV509/2022	B.D.5.2.1.1	GB5.0.5a	PP352487
RSV-B/human/USA/IL-NM-RSV520/2022	B.D.5.2.1.1	GB5.0.5a	PP352492
RSV-B/human/USA/IL-NM-RSV525/2022	B.D.5.2.1.1	GB5.0.5a	PP352490
RSV-B/human/USA/IL-NM-RSV536/2022	B.D.5.2.1.1	GB5.0.5a	PP352491
RSV-B/human/USA/IL-NM-RSV537/2022	B.D.5.2.1.1	GB5.0.5a	PP352493
RSV-B/human/USA/IL-NM-RSV551/2022	B.D.5.2.1.1	GB5.0.5a	PP352486

Supplementary Table 1 | NCBI Accession ID numbers for deposited RSV whole genome sequences.

Step	Primer Sequence
RT	ACGCGAAAAAATGCGTAC
RT	AAYAAAGGAGCATTCAAATA
RT	AAGGKGAACCWATAATAAATT
RT	TATACTATGTMAACAAGCTG
RT	TATATTATGTAAATAAGCAAG
RT	GACCATWGAAGCYATATCA
PCR - Forward (pair 1)	ACGCGAAAAAATGCGTACWAC
PCR - Forward (pair 2)	GCCACARAGTCAATTYATAGTAG
PCR - Forward (pair 3A & B)	TGATGCATCAATATCTCAAGTC
PCR - Forward (pair 4)	GAGATATGCARTTYATGAGYA
PCR - Reverse (pair 1)	TTTGATTGMAAAWCGTGTAGCTG
PCR - Reverse (pair 2)	TGTRACTGGTGTGYTTYTGG
PCR - Reverse (pair 3A)	AGGACTTTCTTTATACTAGCTG
PCR - Reverse (pair 3B)	AGGACTTTTTTGATACTGGCTG
PCR - Reverse (pair 4)	TGRATTTAAACTTATTCTTCCTAGA

Supplementary Table 2 | Primers for RT-PCR Used in the RSV Sequencing Pipeline.
