# Supplementary material for: Deviations in RSV epidemiological patterns and population structures in the United States following the COVID-19 pandemic
Source: Nat Commun. 2024 Apr 20;15:3374. doi: 10.1038/s41467-024-47757-9 (PMC11032338; doi:10.1038/s41467-024-47757-9)
Supplement: Supplementary file 3 — Reporting Summary [file 41467_2024_47757_MOESM3_ESM.pdf]

nature portfolio

Corresponding author(s): Judd F. Hultquist, Estefany Rios-Guzman

Last updated by author(s): 04/02/2024

Reporting Summary

Nature Portfolio wishes to improve the reproducibility of the work that we publish. This form provides structure for consistency and transparency in reporting. For further information on Nature Portfolio policies, see our Editorial Policies and the Editorial Policy Checklist.

Statistics

For all statistical analyses, confirm that the following items are present in the figure legend, table legend, main text, or Methods section.

n/a	Confirmed	
☐	☒	The exact sample size (*n*) for each experimental group/condition, given as a discrete number and unit of measurement
☐	☒	A statement on whether measurements were taken from distinct samples or whether the same sample was measured repeatedly
☐	☒	The statistical test(s) used AND whether they are one- or two-sided<br>*Only common tests should be described solely by name; describe more complex techniques in the Methods section.*
☐	☒	A description of all covariates tested
☐	☒	A description of any assumptions or corrections, such as tests of normality and adjustment for multiple comparisons
☐	☒	A full description of the statistical parameters including central tendency (e.g. means) or other basic estimates (e.g. regression coefficient) AND variation (e.g. standard deviation) or associated estimates of uncertainty (e.g. confidence intervals)
☐	☒	For null hypothesis testing, the test statistic (e.g. *F*, *t*, *r*) with confidence intervals, effect sizes, degrees of freedom and *P* value noted<br>*Give P values as exact values whenever suitable.*
☐	☒	For Bayesian analysis, information on the choice of priors and Markov chain Monte Carlo settings
☒	☐	For hierarchical and complex designs, identification of the appropriate level for tests and full reporting of outcomes
☒	☐	Estimates of effect sizes (e.g. Cohen's *d*, Pearson's *r*), indicating how they were calculated

Our web collection on statistics for biologists contains articles on many of the points above.

Software and code

Policy information about availability of computer code

Data collection

Code for all figures, tree files, BEAST XML files, BEAST log files, and raw data are available at https://github.com/erg6437/RSV-Molecular-Epidemiology. Individualized clinical data cannot be made publicly available in compliance with IRB protocol.

Data analysis

Statistical analyses were performed in R version 4.2.2 and Python version 3.9.5. All multivariable logistic regressions were performed with the glm base function including all indicated variables using p-value<0.05 to assign statistically significant variables associated with differences between RSV-A and RSV-B cases.

The following packages for data analysis are as follows:
Python Dependencies
Pandas (v.1.1.3)
Numpy (v.1.19.2)
matplotlib (v.3.3.2)
seaborn (v.0.11.0)
tableone (v.0.7.12)
bio (v.1.79)
scipy (v.1.5.2)
lifelines (v.0.27.7)
R Dependecies
tabplot
readxl (v.1.4.3)
dplyr (v.1.1.2)

lubridate (v.1.9.2)
ggplot2 (v.3.4.3)
tidyr (v.1.3.0)
ggsci (v.3.0.0)
ggpubr (v.0.6.0)
FastaUtils
ape (v.5.7-1)
TreeTools (v.1.10.0)
phylotools (v.1.9-16)
gtsummary (v.1.7.2)
MASS (v.7.3-58.2)
car (v.3.1-2)
gt (v.0.9.0)
OddsPlotty (v.1.0.2)
effectsize (v.0.8.5)

Alignment and Phylogenetic Reconstruction:
mafft (v7.490)
iqtree2(v2.2)
treetime (0.8.5)

Hypothesis Testing Using Phylogenies (HyPhy) v.2.4.0 was used to execute both a mixed-effects model of evolution (MEME) and a Fast, Unconstrained Bayesian AppRoximation (FUBAR) to determine statistically significant sites undergoing positive selection, as measured by using p-value<0.05 and posterior probability of positive selection > 0.900, respectively.

For manuscripts utilizing custom algorithms or software that are central to the research but not yet described in published literature, software must be made available to editors and reviewers. We strongly encourage code deposition in a community repository (e.g. GitHub). See the Nature Portfolio guidelines for submitting code & software for further information.

Data

Policy information about availability of data

All manuscripts must include a data availability statement. This statement should provide the following information, where applicable:

- Accession codes, unique identifiers, or web links for publicly available datasets
- A description of any restrictions on data availability
- For clinical datasets or third party data, please ensure that the statement adheres to our policy

National epidemiological data obtained from NREVSS is available at https://data.cdc.gov/Laboratory-Surveillance/Respiratory-Syncytial-Virus-Laboratory-Data-NREVSS/52kb-ccu2/about_data and hospitalization data from RSV-NET is available at https://data.cdc.gov/Public-Health-Surveillance/Weekly-Rates-of-Laboratory-Confirmed-RSV-Hospitali/29hc-w46k/about_data. Data extending beyond from June 27th, 2020 to June 3rd 2023 were not available in this dataset, so the data were consequently accessed in the CDC NREVSS National Trends (https://www.cdc.gov/surveillance/nrevss/rsv/natl-trend.html). Chicago Department of Public Health (CDPH) testing, case count, and percent positivity data were provided by the Vaccine-Preventable Disease Surveillance Program. Consensus sequences assembled by our group have been deposited on GenBank with respective accession numbers listed in supplementary table 1. All other accession numbers for publicly available RSV WGS is listed in our GitHub repository: https://github.com/erg6437/RSV-Molecular-Epidemiology/tree/main/NCBI-Information. For the clinical dataset used in this study, the IRB restricts the publishing or release of individualized clinical datasets. Therefore, this is the only dataset which is not available for the public and the peer review process. However, any requests for aggregated and de-identified datasets can be made to judd.hultquist@northwestern.edu within 3 years of publication. The amount and format of the requested data that can be shared are dictated by the data-sharing agreements and IRB protocols of each respective institution.

Research involving human participants, their data, or biological material

Policy information about studies with human participants or human data. See also policy information about sex, gender (identity/presentation), and sexual orientation and race, ethnicity and racism.

Reporting on sex and gender	The sex of the patient encounters was incorporated into the logistic regression analysis as a variable when identifying the relationship between clinical outcome and RSV subtype. Within our clinical dataset, information of biological sex is provided; we do not have access to, nor is there self-reporting of gender. In this analysis, we aggregate our clinical data and do not expand on individual-level data.
Reporting on race, ethnicity, or other socially relevant groupings	The population level characteristics used in the study included race, and ethnicity. This metadata was stored and pulled from Northwestern Medicine (NM)'s electronic data warehouse (EDW) repository. Race and ethnicity are self-reported and these variables are not used as proxies for social construct outcomes. These socially relevant variables were used in the logistical analysis to better elucidate the relationship between clinical outcomes and RSV genetic diversity.
Population characteristics	Other population-level and covariate information included sex, age, race, BMI, sum of comorbidities (i.e. hypertension, cancer, coronary artery disease, renal disease, diabetes, asthma, liver disease, immunocompromised status, stem cell transplant, solid organ transplant, and HIV status), admission status, and outcome data. Our cohort is comprised of individuals averaging the age of 60+ who were hospitalized in Northwestern Medicine-affiliated institutions typically reported with several of the co morbidities listed above. These metrics are reported in our Demographics Table (Supp. Table 1).
Recruitment	The clinical isolates collected for the study are from residual diagnostic tests. Therefore, there was no recruitment process was involved for our study. Patient encounters were reflective of Northwestern Medicine's patient demographics and may an

origin of self-selection bias. Consequently, the demographic makeup of our patient encounter cohort may be skewed towards populations seeking medical attention at Northwestern Medicine-affiliated institutions.

Ethics oversight	The Northwestern University institutional review board (IRB) approved the study protocols for this study that included both the residual diagnostic specimen collection and the clinical metadata collection (#STU00212260, #STU00206850, and #STU00207123).

Note that full information on the approval of the study protocol must also be provided in the manuscript.

Field-specific reporting

Please select the one below that is the best fit for your research. If you are not sure, read the appropriate sections before making your selection.

☒ Life sciences ☐ Behavioural & social sciences ☐ Ecological, evolutionary & environmental sciences

For a reference copy of the document with all sections, see nature.com/documents/nr-reporting-summary-flat.pdf

Life sciences study design

All studies must disclose on these points even when the disclosure is negative.

Sample size	No sample size calculation was used to determine our cohort of RSV-positive patient encounters. We utilized all patient encounters from our study period (January 2009 to February 2023) in our study and subsequently included inpatient encounters for clinical analysis as per IRB guidelines.
Data exclusions	Exclusion criteria for the clinical metadata was used to exclude all pediatric inpatient encounters, adult outpatient encounters, and pediatric outpatient encounters for clinical analysis to abide by IRB study protocol. Patient encounter with demographic data missingness (i.e., age, sex, race, ethnicity) were additionally excluded from regression modeling. Clinical isolates with insufficient viral load or incomplete whole genome amplification were excluded from the phylogenetic analysis portion of the study.
Replication	No analysis was conducted in this study that required replicates or replication-like methods for the clinical metadata. Replication of the clinical analysis was not performed, as the clinical outcomes are terminal time points and there were no interventions or change in standard-of-care for these patients. Both bayesian and maximum likelihood (ML) phylogenetic analysis included use of multiple Markov chain Monte Carlo (MCMC) runs or likelihood-ratio test replicates, respectively. No other experiments were performed that involved use of replicates.
Randomization	No randomization was used in this study. Allocation of experimental groups (i.e. RSV-A vs RSV-B) was based on subtyping information provided by previous diagnostic testing information or in-house PCR-based typing protocols.
Blinding	Blinding was not applicable to this study because our analysis retroactively collects clinical metadata and isolates which are then analyzed on a population-wide scale. Since there were no group intervention or drug/treatment allocation, testing and validation of the dataset was not performed. Consequently, the investigators were not blinded to the patient groupings as there were no patient allocations.

Reporting for specific materials, systems and methods

We require information from authors about some types of materials, experimental systems and methods used in many studies. Here, indicate whether each material, system or method listed is relevant to your study. If you are not sure if a list item applies to your research, read the appropriate section before selecting a response.

Materials & experimental systems

n/a	Involved in the study	
☒	☐	Antibodies
☒	☐	Eukaryotic cell lines
☒	☐	Palaeontology and archaeology
☒	☐	Animals and other organisms
☐	☒	Clinical data
☒	☐	Dual use research of concern
☒	☐	Plants

Methods

n/a	Involved in the study	
☒	☐	ChIP-seq
☒	☐	Flow cytometry
☒	☐	MRI-based neuroimaging

Clinical data

Policy information about clinical studies

All manuscripts should comply with the ICMJE guidelines for publication of clinical research and a completed CONSORT checklist must be included with all submissions.

Clinical trial registration	NA - this study did not involve a clinical trial
Study protocol	The collection of RSV-positive residual diagnostic specimens for viral whole genome sequencing was approved through IRB

Study protocol	#STU00212260 and #STU00206850. Clinical and demographic data is stored on a secure Research Electronic Data Capture (REDCap) server in compliance with HIPAA regulations following IRB #STU00207123.
Data collection	Demographic and clinical metadata from RSV+ patient encounters were extracted from NM’s enterprise data warehouse (EDW). A biobank of RSV diagnostic specimens was formed under the Center of Pathogen Genomics and Microbial Evolution (CPGME) Center at Northwestern University. Residual diagnostic nasopharyngeal swabs from patients with a confirmed positive RSV infection in the Northwestern Medicine healthcare system were collected from December 17, 2017, through January 1, 2023.
Outcomes	Discharge and death dates used as our outcomes-of-interest are defined in Northwestern Medicine's EDW. An RSV-associated death is defined as a death upon 30 days of admission to the hospital.

Plants

Seed stocks	NA - no plants were used in this study
Novel plant genotypes	NA - no plants were used in this study
Authentication	NA - no plants were used in this study
